# Radioembolization for Colorectal Liver Metastases: Indications and Outcomes

**DOI:** 10.1007/s12029-026-01617-5

**Published:** 2026-10-27

**Authors:** Sai Reddy, Xiao Wu, Natasha Rodwell, Abdul Khan, Rakesh Navuluri, Virginia Shaffer, Nicholas Fidelman, Qian Yu

**Affiliations:** 1 University of Chicago Pritzker School of Medicine, Chicago, IL USA; 2 Department of Vascular & Interventional Radiology, University of California San Francisco, San Francisco, CA USA; 3 Department of Vascular & Interventional Radiology, University of Chicago, 5841 S Maryland Ave, Chicago, IL 60637 USA; 4 Department of Colon & Rectal Surgery, University of Chicago, Chicago, IL USA

**Keywords:** Radioembolization, Selective Internal Radiation Therapy (SIRT), Colorectal cancer, Liver metastases, Transarterial Radioembolization (TARE), Y-90

## Abstract

Colorectal cancer remains a leading cause of cancer-related mortality, with the liver representing the predominant site of metastatic spread. For patients with colorectal liver metastases (CRLM) who are not candidates for surgical resection, systemic chemotherapy provides disease control but rarely achieves durable hepatic responses. Radioembolization with yttrium-90 (Y-90) microspheres, delivered via the hepatic artery to exploit the preferential arterial supply of liver tumors, has emerged as an important locoregional option in this setting. This review examines the current evidence base for radioembolization in CRLM, covering patient selection, indications across lines of therapy, dosimetric advances, and clinical outcomes. Appropriate candidacy requires careful assessment of performance status, hepatic tumor burden, tumor biology, lung shunt fraction, and extrahepatic disease extent, with the strongest outcomes observed in patients selected earlier in their disease course. Randomized controlled trials support second-line and third-line use, while unselected first-line use has not demonstrated a benefit. Dosimetric advances, including multicompartment personalized dosing, radiation segmentectomy, and radiation lobectomy, are enabling ablative and bridge-to-resection strategies in appropriately selected patients. Future investigation should prioritize biomarker-driven patient selection, personalized dosimetry, and combination strategies with systemic therapy to determine whether durable hepatic disease control can translate into a survival benefit.

## Introduction

 Colorectal cancer (CRC) remains a major contributor to cancer-related morbidity and mortality in the United States, ranking as the second leading cause of cancer death and the leading cause among adults under 50 years of age [1]. The liver is the predominant site of hematogenous spread, with approximately 25% of CRC patients presenting with hepatic involvement at the time of diagnosis, and up to 60% developing liver metastases over their disease course [2]. Despite advances in systemic therapy, colorectal liver metastases (CRLM) continue to carry a poor prognosis. Untreated, median survival is less than 9 months and 5-year survival rate, at best, is 11% [3, 4].

Surgical resection is a potentially curative treatment for CRLM, with 5-year overall survival (OS) rates of 30–50% in appropriately selected patients [5]. Unfortunately, only 10–25% of patients with CRLM are candidates for upfront resection [6]. For the majority, management relies on systemic chemotherapy, including oxaliplatin- and irinotecan-based regimens combined with targeted biologic agents. This method has meaningfully extended survival but rarely achieved durable disease control [7]. Thermal ablation, stereotactic body radiotherapy (SBRT), irreversible electroporation, and histotripsy provide additional local treatment options for patients with limited, technically accessible lesions, though each is constrained by tumor size, location, and the number of treatable lesions [8, 9].

For patients with liver-dominant disease who are not appropriate candidates for resection or ablation, locoregional therapies delivered via the hepatic arterial system have emerged as an important component of the multidisciplinary treatment algorithm. Since metastatic tumors greater than 3 mm are supplied primarily by the hepatic artery, treatments administered through this route enhance delivery of tumoricidal agents directly to tumor tissue while limiting systemic and hepatic toxicity [10]. Among these approaches, radioembolization with Yttrium-90 (Y-90) microspheres, also called selective internal radiation therapy (SIRT) or transarterial radioembolization (TARE), has evolved from a palliative sequential lobar approach in the salvage setting to an increasingly precise, ablative treatment strategy. This review examines the role of radioembolization in the management of CRLM, with a focus on patient selection, indications, and clinical outcomes data from key retrospective and prospective studies.

## Radioembolization: Mechanism, Procedure, and Rationale

Radioembolization is a form of transcatheter brachytherapy in which radioactive microspheres are delivered directly into the hepatic artery to treat primary and secondary liver tumors. The procedure capitalizes on the preferential delivery of microspheres to the tumor capillary bed, allowing high doses of radiation to be concentrated within tumor tissue relative to the non-tumorous hepatic parenchyma [11]. The radioactive isotope used is Y-90, a beta emitter that has a mean tissue penetration range of 2.5 mm and a half-life of 64.2 h. This combination of short range and predictable decay makes Y-90 well-suited to liver-directed therapy to deliver a concentrated tumoricidal dose while limiting local and systemic radiation exposure [12].

**Fig. 1:**
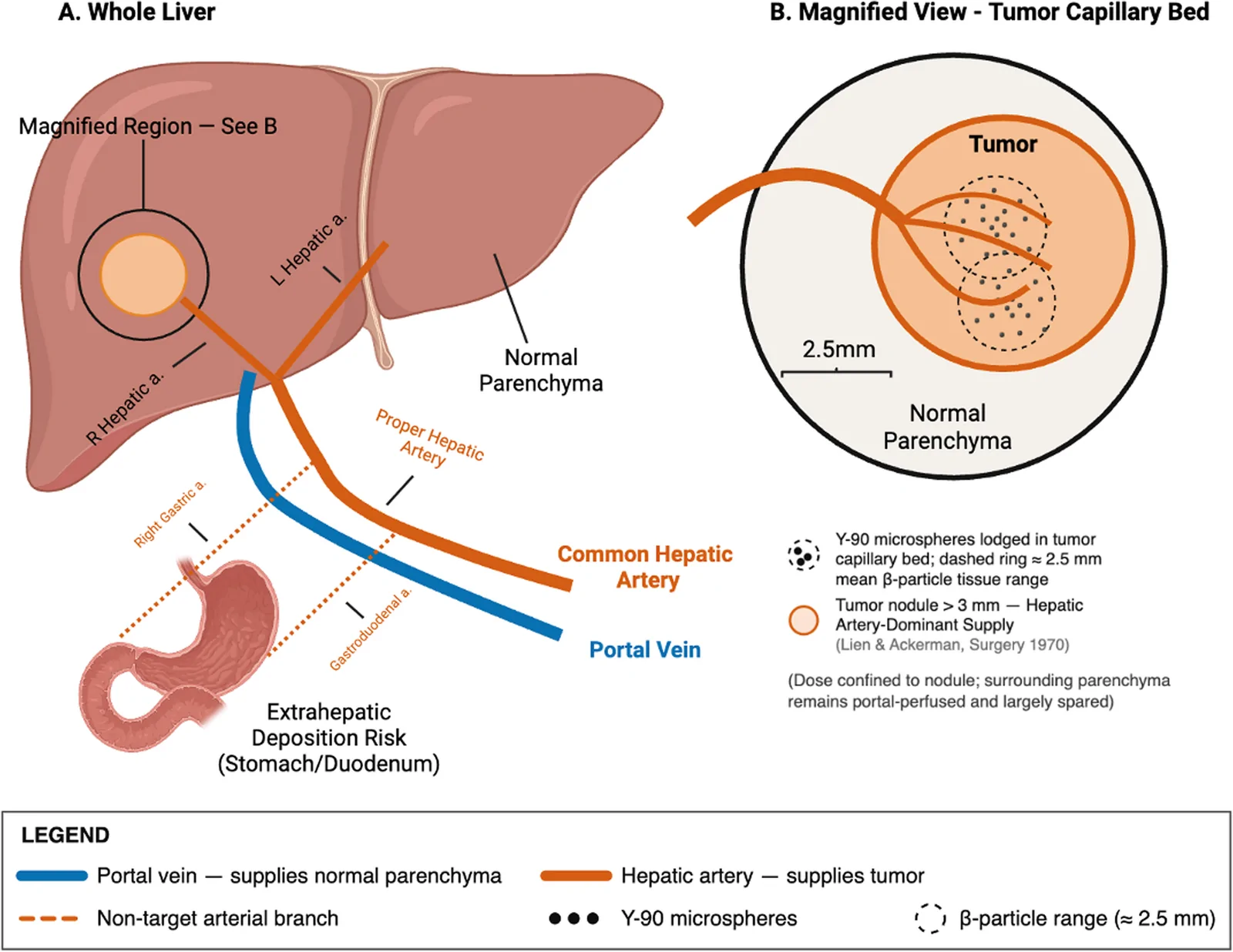
Dual hepatic blood supply and the basis for selective intra-arterial Y-90 delivery. (**A**) Whole-liver schematic demonstrating the liver’s dual vascular supply: the portal vein (blue) perfuses normal parenchyma, while hepatic artery divisions (orange) preferentially supply tumor deposits, consistent with the arterial-dominant flow (80–100%) demonstrated for CRLM > 3 mm. Non-target arterial branches arising proximal to the liver — the gastroduodenal and right gastric arteries — are shown coursing toward the stomach and duodenum, marking sites of potential extrahepatic microsphere deposition. (**B**) Magnified view of the boxed region in (A), showing the tumor capillary bed. Y-90 microspheres (black dots) lodge within the tumor nodule following intra-arterial delivery; dashed rings indicate the ≈ 2.5 mm mean tissue range of emitted β-particles, illustrating how dose remains largely confined to the tumor while the portal-perfused surrounding parenchyma is spared

The biological rationale for hepatic arterial delivery is well established and visually depicted in Fig. 1. While normal hepatic parenchyma receives the majority of its blood supply from the portal vein, metastatic liver tumors derive their blood supply from the hepatic artery to augment blood flow to the tumor growth site [13]. This differential vascular supply is the foundational component of radioembolization’s therapeutic selectivity, with microspheres injected into the hepatic artery to preferentially target hypervascular tumor tissue while limiting radiation-mediated damage to functional hepatic reserve. The perfusion source can be quantified noninvasively on standard pretreatment triphasic computed tomography (CT) using the arterial enhancement fraction (AEF), the ratio of arterial-phase to portal-phase tumor enhancement. In a retrospective series of 60 CRLM, AEF was significantly higher in tumors that achieved a metabolic response to radioembolization than in nonresponders (*P* = 0.038), with response rates of 40% for tumors with an AEF < 0.4 vs. 78% for AEF > 0.75 (Boas 2016). This study proposes AEF as an early imaging predictor of hepatic artery-dominant tumor perfusion and radioembolization responsiveness.

**Fig. 2:**
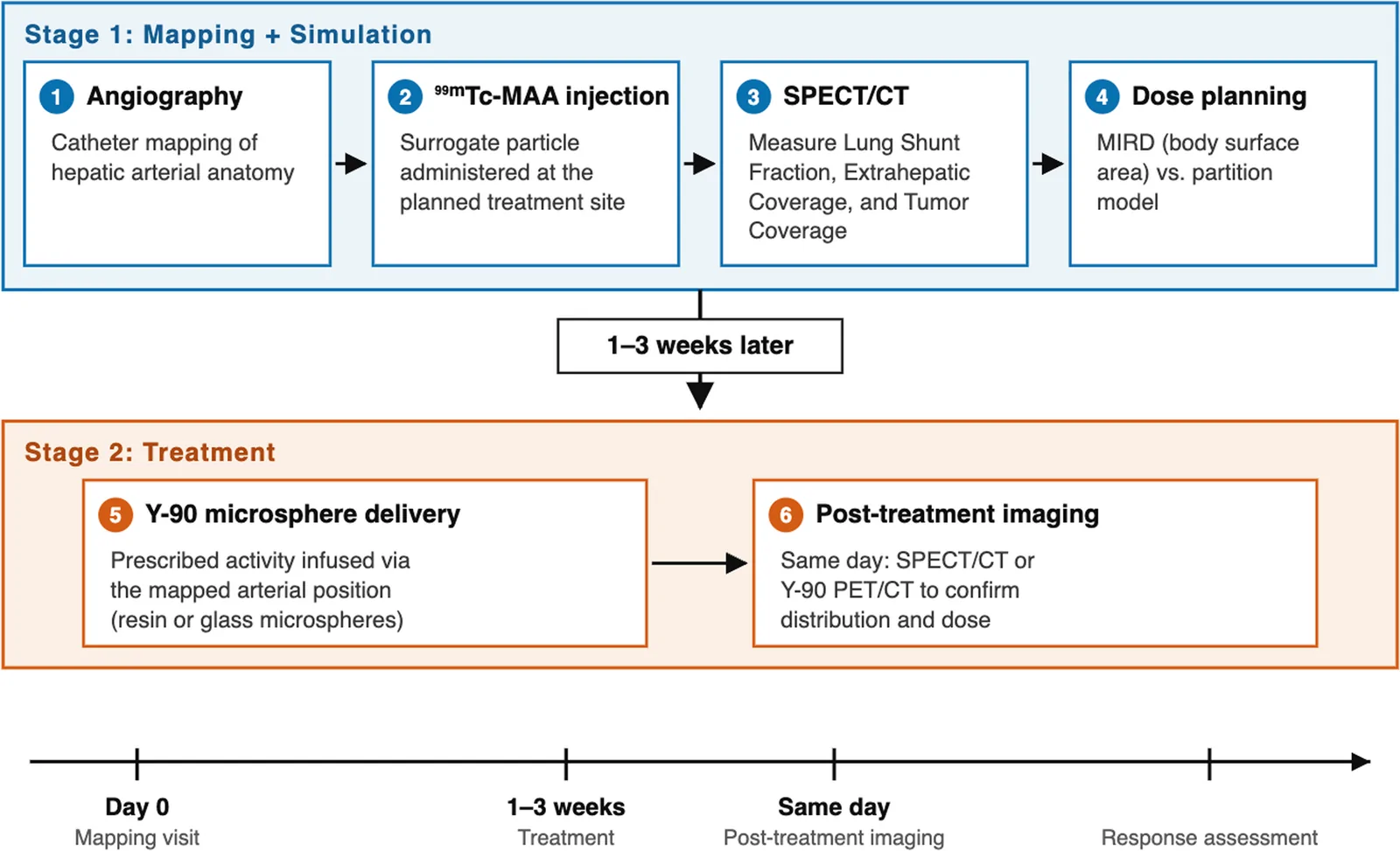
Two-stage Y-90 radioembolization workflow and typical intervals. At the mapping visit, hepatic angiography defines the arterial anatomy. ^99m^Tc-MAA is then injected at the intended treatment position and single-photon emission CT (SPECT/CT) is used to quantify LSF, detect extrahepatic deposition, and estimate tumor coverage before dose planning. Treatment follows after an interval of 1–3 weeks, with same-day post-treatment SPECT/CT or Y-90 positron emission tomography (PET)/CT to confirm microsphere distribution

Radioembolization is performed as a two-stage procedure, depicted in Fig. 2. The first stage is the planning/mapping visit occurring 1–3 weeks prior to treatment. During this visit, a diagnostic angiogram is performed to evaluate hepatic arterial anatomy, including any variant vessels such as replaced or accessory hepatic arteries. An important goal of the mapping procedure is to identify the arterial supply to the liver tumor(s) and distinguish it from vessels supplying non-target structures. If the risk of nontarget embolization is high, arteries supplying non-target structures, such as the gastroduodenal, right gastric, supraduodenal, and cystic arteries, may require prophylactic embolization to prevent inadvertent microsphere deposition in the gastrointestinal tract. During mapping, technetium-99 mm macroaggregated albumin (^99m^Tc-MAA) is administered intra-arterially from the planned treatment position. Because ^99m^Tc-MAA mimics the embolic behavior of Y-90 microspheres, their subsequent distribution on scintigraphic imaging serves three critical purposes: quantification of the lung shunt fraction (LSF), identification of any extrahepatic deposition, and confirmation of adequate tumor coverage to guide dosimetric planning [14, 15]. The mapping step also informs dose selection. Current dosing methodology has moved away from the empiric body surface area (BSA) model toward mean absorbed-dose approaches since the BSA model does not account for liver volume and tumor burden. Two models are favored: the medical internal radiation dose (MIRD) model and the partition model. The MIRD model assumes homogenous activity distribution across the treated angiosome (tumor and non-tumor tissue together) and therefore does not distinguish tumor dose from normal liver dose [16]. The partition dosimetry model, though more resource-intensive, estimates tumor and non-tumor compartment doses separately using the ^99m^Tc-MAA -derived tumor-to-normal ratio, enabling tumor dose to be maximized while constraining normal liver tissue exposure. Model selection is therefore driven by treatment volume. The partition model is favored for large-volume lobar treatment, where maximizing tumor dose while protecting the surrounding parenchyma is the governing constraint. For small-volume, highly selective treatment such as radiation segmentectomy, the single-compartment MIRD model is favored, since the normal liver within the target segment is itself expected to receive an ablative dose [16, 17]. The second stage of the treatment involves delivery of the Y-90 microspheres via the target hepatic arteries identified during mapping, followed by post-treatment imaging to confirm microsphere distribution and dose delivery estimation.

## Patient Selection & Indications

Appropriate patient selection is crucial to optimize outcomes and minimize toxicities from radioembolization. Standard inclusion criteria for a Y-90 radioembolization require an Eastern Cooperative Oncology Group (ECOG) performance status of 0–2, a life expectancy of at least 3 months, and liver-dominant disease [18].

### Candidacy Criteria

There are several selection parameters that emerge consistently across the literature as the most clinically meaningful determinants of outcome. In a landmark retrospective multicenter study, Hickey et al. analyzed 531 patients with CRLM treated with TheraSphere glass microspheres across 8 institutions between 2001 and 2014. On multivariate analysis, ECOG performance status of 0 (HR 0.61), hepatic tumor burden ≤ 25% (HR 0.37), absence of extrahepatic disease (HR 0.50), albumin > 3 g/dL (HR 0.47), and receipt of two or fewer prior cytotoxic regimens (HR 0.61) independently predicted improved survival [19]. Notably, tumor burden carried the strongest effect size of the five variables, suggesting that patient selection earlier in the disease course, before extensive hepatic replacement occurs, is the single most impactful modifiable determinant of improved survival. Consistent with these findings, exploratory subgroup analyses of the EPOCH phase III randomized controlled trial found that patients with KRAS mutations, ECOG 0, baseline carcinoembryonic antigen (CEA) ≤ 35 ng/mL, and absence of extrahepatic disease derived the greatest benefit in progression-free survival (PFS) and hepatic PFS from TARE plus second-line chemotherapy [20]. In terms of intrahepatic tumor burden, international multidisciplinary consensus recommendations propose a maximum acceptable threshold of approximately 50% replacement, above which the risk of toxicity and/or rapid disease progression rises and treatment becomes a relative contraindication [21, 22]. Where extrahepatic disease is present, candidacy may still be considered if burden is limited, generally defined as ≤ 10 pulmonary nodules and either lymph node involvement confined to one region or one additional metastatic site amenable to future definitive treatment [22]. This threshold has been applied prospectively by Fidelman et al., who conducted a phase I trial of TARE combined with trifluridine/tipiracil (TAS-102) in chemorefractory liver-dominant metastatic colorectal cancer (mCRC) [23]. This study required tumor replacement < 50% of total liver volume and excluded patients with symptomatic extrahepatic disease, > 10 pulmonary nodules, or peritoneal carcinomatosis. Notably, 67% of enrolled patients still experienced asymptomatic low-volume extrahepatic disease at baseline, and 62% progressed extrahepatically at a median of 3.6 months, suggesting that permitting limited extrahepatic disease may itself have diminished the detectable benefit of hepatic-directed therapy [23]. These burden-based thresholds are further corroborated by Damm et al., who analyzed 106 chemotherapy-refractory CRLM patients and developed the composite TuCK prognostic score, assigning one point each for tumor load > 20%, Karnofsky index < 80%, and CEA > 130 ng/mL or CA19-9 > 200 U/mL. Patients scoring 0–1 achieved a median OS of 10.4 months compared to 5.1 months for those scoring 2–3 (*P* < 0.001), reinforcing that radioembolization in a heavily burdened, poor-performance population carries limited benefit and should be approached with restraint [24]. Tumor biology independent of burden also predicts outcome. In a single-center analysis of 53 heavily pretreated salvage patients, CEA ≥ 90 ng/mL at the time of radioembolization and lymphovascular invasion on primary tumor pathology were independent predictors of shorter OS (7.1–7.2 vs. 16.9 months for each factor, respectively), while other established markers of burden (25% liver replacement, synchronous disease, bilobar involvement) were significant only on univariate analysis [25]. These findings suggest that tumor biology markers such as lymphovascular invasion may carry as much prognostic weight as anatomic tumor burden, and warrant incorporation into future prospective selection criteria. Taken together, the strongest candidates for radioembolization in CRLM are patients with preserved performance status (ECOG 0), hepatic tumor burden below approximately 50%, favorable tumor biology (KRAS wild-type, lower CEA, absence of lymphovascular invasion), and either no extrahepatic disease or limited, controlled extrahepatic disease amenable to future local treatment.

### Contraindications

Contraindications to radioembolization can be stratified into absolute and relative, and many are identified during the pre-treatment mapping procedure. Absolute contraindications include uncorrectable hepato-enteric arterial communications (e.g., stomach, duodenum, or bowel) that cannot be excluded from the treatment zone via microsphere delivery vascular site selection or prophylactic coil embolization, as inadvertent microsphere deposition in these territories carries the risk of gastrointestinal ulceration or perforation [26]. The second absolute contraindication relates to hepatopulmonary shunting. Although the resin microsphere package insert specifies graded activity reduction for a LSF above 10%, this is not reflected in contemporary practice. For both resin and glass microspheres, the operative limit is dosimetric rather than shunt based. Specifically, an estimated lung absorbed dose > 30 Gy from a single administration or > 50 Gy over a lifetime are regarded as absolute contraindicated due to the increased risk of radiation pneumonitis [27]. Relative contraindications, apart from poor performance status and tumor burden explained earlier, include severe portal hypertension with compromised portal venous flow, prior hepatic radiation, limited pulmonary reserve, and extensive extrahepatic disease where hepatic progression is not the dominant clinical problem. Notably, in Hickey et al.’s retrospective study, no cases of gastrointestinal ulceration were observed, supporting an emerging practice of selective rather than routine prophylactic coil embolization of at-risk vessels in experienced centers [19].

## Outcomes

The clinical evidence base for radioembolization in CRLM spans nearly three decades, and its development ran opposite to the treatment continuum. The earliest studies enrolled heavily pretreated patients with no remaining systemic options. Later, randomized phase III trials tested the modality earlier in the disease course. The following summarizes outcomes by line of therapy rather than chronologically, followed by emerging evidence from ablative dosing strategies that represent the next frontier of the field.

### First-Line Setting

Interest in first-line radioembolization was initially motivated by two smaller randomized trials pairing Y-90 microspheres with first-line chemotherapy. In a trial of Y-90 resin microspheres plus hepatic arterial chemotherapy with floxuridine (FUDR) versus FUDR alone, the addition of SIRT more than doubled objective response rates and reduced the risk of death from hepatic progression roughly threefold [28]. A subsequent trial randomizing 21 patients to fluorouracil/leucovorin with or without Y-90 resin microspheres reported a striking OS difference (29.4 vs. 12.8 months, HR 0.33) [29]. These early, hypothesis-generating signals motivated three large, prospective randomized phase III trials designed to test the combination treatment at scale: SIRFLOX, FOXFIRE, and FOXFIRE-Global. Each of these studies evaluated the combination of FOLFOX6m plus Y-90 resin microspheres versus FOLFOX6m alone in patients with liver-only or liver-dominant non-resectable CRLM, with limited extrahepatic disease in the lung or lymph nodes. Across all three trials, oxaliplatin dose was reduced from 85 mg/m² to 60 mg/m² for the cycle coinciding with SIRT and the two subsequent cycles, a modification informed by earlier phase I data establishing 60 mg/m² as the maximum tolerated oxaliplatin dose with concurrent SIRT, given dose-limiting neutropenia at full-dose FOLFOX4 [30]. In a combined analysis of 1,103 patients across all three trials, the addition of radioembolization to first-line chemotherapy increased the objective response rate from 63% to 72% (OR 1.52, 95% CI 1.18–1.96; *P* = 0.0012), with a larger effect on liver-specific response (OR 1.78, 95% CI 1.37–2.31; *P* < 0.0001), and approximately halved the risk of hepatic progression (HR 0.51, 95% CI 0.43–0.62, *P* < 0.001). However, neither overall PFS (11.0 vs. 10.3 months; *P* = 0.11) or OS (22.6 vs. 23.3 months; *P* = 0.61) differed between arms, likely due to extrahepatic disease progression [31]. The null result may nonetheless mask heterogeneity by primary tumor sidedness. In a combined analysis of SIRFLOX and FOXFIRE-Global involving 739 patients, the addition of SIRT conferred no survival benefit in the full cohort (24.3 vs. 24.6 months; *P* = 0.81), but among the 179 patients with right-sided primary tumors, median OS improved from 17.1 to 22.0 months (HR 0.64, *P* = 0.008). No corresponding benefit was seen among the 540 patients with left-sided primaries (24.6 vs. 26.6; HR 1.12: *P* = 0.264) [32]. One proposed explanation is the lower chemosensitivity of right-sided colon cancer, which may have increased the relative contribution of liver-directed therapy in this subgroup. These findings established that unselected first-line use of radioembolization alongside chemotherapy cannot be justified on the basis of survival benefit alone, and refocused investigational interest on better-defined patient subgroups and later lines of therapy.

### Second-Line Setting

That refocusing produced the pivotal phase III EPOCH trial, the most significant advancement in the field to date. EPOCH enrolled 428 patients with unresectable unilobar or bilobar CRLM who had progressed on first-line oxaliplatin- or irinotecan-based therapy, randomized 1:1 to Y-90 glass TARE plus standard-of-care second-line chemotherapy (*n* = 215) versus chemotherapy alone (*n* = 213). Treatment with TARE and chemotherapy resulted in a median PFS of 8.0 months compared to 7.2 months with chemotherapy alone (HR 0.69, *P* = 0.0013), and a median hepatic PFS of 9.1 months versus 7.2 months (HR 0.59, *P* < 0.0001). Objective response rates were 34.0% in the TARE group versus 21.1% in the chemotherapy-alone group (*P* = 0.0019). However, median OS was similar between arms at 14.0 and 14.4 months respectively (*P* = 0.7229). Despite the absence of an OS benefit, the addition of TARE to second-line chemotherapy extended PFS (8.0 vs. 7.2 months; HR 0.69; 95% CI 0.54–0.88; *P* = 0.0013), hepatic PFS (9.1 vs. 7.2 months; HR 0.59; 95% CI, 0.46–0.77; *P*< 0.0001, and time to subsequent treatment (21.0 vs. 10.1 months; HR 0.49; 95% CI 0.37–0.67) without compromising quality of life [21, 33]. The EPOCH trial established a formal evidence basis for TARE use with second-line systemic therapy. Second-line benefit is echoed in real-world data: in the multicenter RESIN registry, patients treated with TARE in the second-line setting achieved a median OS of 17.4 months, closely aligned with EPOCH’s second-line population and supporting the generalizability of the trial’s findings outside the clinical trial setting [21].

### Salvage and Chemorefractory Setting

Although radioembolization now carries evidence-based support in the first- and second-line settings, its oldest and deepest evidence base remains in salvage therapy, where it was used exclusively for patients who had exhausted two or more lines of systemic chemotherapy and had few remaining options. The first prospective multicenter evaluation of radioembolization monotherapy in this context was conducted by Cosimelli et al., who prospectively enrolled 50 oxaliplatin- and irinotecan-refractory CRLM patients across multiple Italian centers, 76% of whom had received four or more prior lines of chemotherapy and 70% of whom had bilobar hepatic disease. By intention-to-treat analysis, the confirmed objective response rate was 24% by RECIST criteria, including one complete and eleven partial responses, with an additional 24% achieving stable disease. Median OS was 12.6 months (95% CI 7.0–18.3) and 2-year survival was 19.6%. These results were considered meaningful given the extreme pretreatment burden of the cohort, and established the first prospective benchmark for SIRT monotherapy outcomes [34].

In a parallel effort, Hendlisz et al. conducted the first phase III trial in this setting, randomizing 46 patients with liver-limited mCRC refractory to standard chemotherapy to protracted intravenous fluorouracil infusion alone or combined with Y-90 resin microspheres. Though not powered for OS, the combination arm demonstrated a significantly extended time to liver progression of 5.5 months versus 2.1 months in the radioembolization and control arms respectively (*P* = 0.003), while median OS did not differ (10.0 vs. 7.3 months; *P* = 0.80) [35]. Crossover to TARE arm was permitted after progression in the control fluorouracil arm, and 10/23 control patients ultimately received it. This degree of crossover substantially limits interpretation of the OS comparison. This trial provided the first randomized evidence that locoregional liver control could be meaningfully extended with radioembolization in a heavily pretreated population, and established that fluoropyrimidines may be successfully used as radiosensitizers [35].

The finding from Hendlisz et al. that fluoropyrimidines could be delivered safely alongside TARE prompted formal phase I evaluation of radiosensitizing regimens in the salvage setting. Cohen et al. conducted a prospective single-center 3 + 3 dose-escalation study of capecitabine (375–1000 mg/m² twice daily) with sequential lobar Y-90 resin microsphere radioembolization in 24 patients with liver-dominant advanced cancer. The maximum tolerated dose was not reached, hematologic events were generally mild, and the most common grade 1–2 non-hematologic toxicities were transient transaminase or alkaline phosphatase elevation (37.5% each). Capecitabine 1000 mg/m² twice daily was recommended for subsequent phase II study [36]. Fidelman et al. subsequently paired lobar Y-90 resin radioembolization with TAS-102 in 21 patients with bilobar liver-dominant mCRC refractory to fluoropyrimidines, oxaliplatin, irinotecan, and bevacizumab. No dose-limiting toxicities occurred, permitting the full approved TAS-102 dose. Severe treatment-related events were cytopenias (48%) and radioembolization-induced liver disease (10%). Disease control within the treated lobes was 100%, with an overall disease control rate of 76% [23]. Together these trials establish that radiosensitizing chemotherapy can be delivered safely with radioembolization in heavily pretreated patients, neither having reached a maximum tolerated dose.

A retrospective analysis of 53 heavily pretreated CRLM patients from a prospectively maintained single-center database reported a median OS of 12.7 months, closely mirroring the survival observed in both Cosimelli’s and Hendlisz’s cohorts and reinforcing a convergent ~ 12–13 month benchmark for salvage-setting radioembolization across independent series [25]. This series also highlighted a methodological limitation relevant across the salvage literature: by RECIST criteria, the majority of patients were classified as having stable disease (80% at 4–8 weeks), a pattern that likely reflects RECIST’s insensitivity to post-treatment necrosis and fibrotic change rather than true lack of response. Metabolic imaging (PERCIST) reclassified the majority of these patients into either response or progression, suggesting that functional imaging may capture treatment effect more accurately than anatomic size-based criteria alone [25].

## Advances in Dosimetry

### Contemporary Dosimetry

Across all lines of therapy, the trials summarized above share a common limitation: reliance on non-personalized dosing. EPOCH, for example, used single-compartment dosimetry targeting a fixed 120 Gy (± 10%) perfused-volume absorbed dose, with no distinction between tumor and normal-tissue absorbed dose [20, 21] This approach, while practical, results in substantial variability in the dose actually delivered to tumor versus normal liver parenchyma from patient to patient. Advances in radiation dosimetry in CRLM are being actively investigated due to improved objective response rates in personalized, targeted SIRT vs. standard, non-specific dosing for patients with advanced hepatocellular carcinoma (HCC) [37]. In 2025, the TheraSphere Global Steering Committee published consensus dosimetry recommendations for this personalized approach in colorectal, neuroendocrine, and cholangiocarcinoma liver metastases. For CRLM, the committee recommends multicompartment dosimetry for standard uni- and bilobar treatment, targeting a normal-tissue absorbed dose ceiling of 75 Gy alongside a tumor absorbed dose of 150–200 Gy [22]. For ablative-intent treatment, the committee recommends a single-compartment perfused-volume absorbed dose of ≥ 400 Gy for radiation segmentectomy and 150 Gy for radiation lobectomy [22]. This dose-escalation is supported by Dimopoulos et al. where one-year local tumor progression-free survival was 68.6% for tumors receiving a mean tumor absorbed dose ≥ 400 Gy, compared to 14.3% for those below this threshold (*P* = 0.013), with dose remaining an independent predictor of local control on multivariate analysis [38].

A parallel set of international recommendations addresses resin microspheres. Levillian et al. endorsed personalized activity prescription using partition and/or voxel-based dosimetry for both whole-liver and selective treatment. For normal parenchyma, a mean absorbed dose to non-tumoral liver of 40 Gy or less was considered safe. For tumor cells, a minimum mean absorbed dose of 100–120 Gy was recommended in CRLM [39]. Consistent with this threshold, a retrospective single-institution study demonstrated that delivering a mean tumor dose of ≥ 100 Gy using resin microspheres was significantly associated with objective tumor response and prolonged OS, providing quantitative evidence that dose escalation translates to meaningful clinical benefit [40]. These thresholds are device-specific and are not interchangeable with those derived for glass microspheres, which differ in specific activity, particle number, and intrahepatic distribution.

### Radiation Segmentectomy

Building on this dosimetric threshold, radiation segmentectomy has emerged as a curative-intent paradigm for patients with limited hepatic disease, particularly those with oligometastatic CRLM not amenable to surgical resection or thermal ablation (Fig. 1). This is a selective approach that was established for HCC [41], but aims to deliver high-dose radiation in CRLM to ≤ two hepatic segments while minimizing damage to surrounding healthy tissue [42]. Long-term data support this ablative approach. A single-institution series applying escalated segmental and modified radiation-lobectomy dosing over a 10-year period reported durable local control (98%) in appropriately selected patients, providing early evidence that escalated dosing and modified radiation segmentectomy can achieve outcomes comparable to other ablative modalities in liver-dominant CRLM [43]. Prospective trials specifically evaluating radiation segmentectomy as a curative alternative in CRLM are needed to define patient selection criteria and comparative efficacy relative to ablation and SBRT in the oligometastatic setting.

### Radiation Lobectomy

**Fig. 3:**
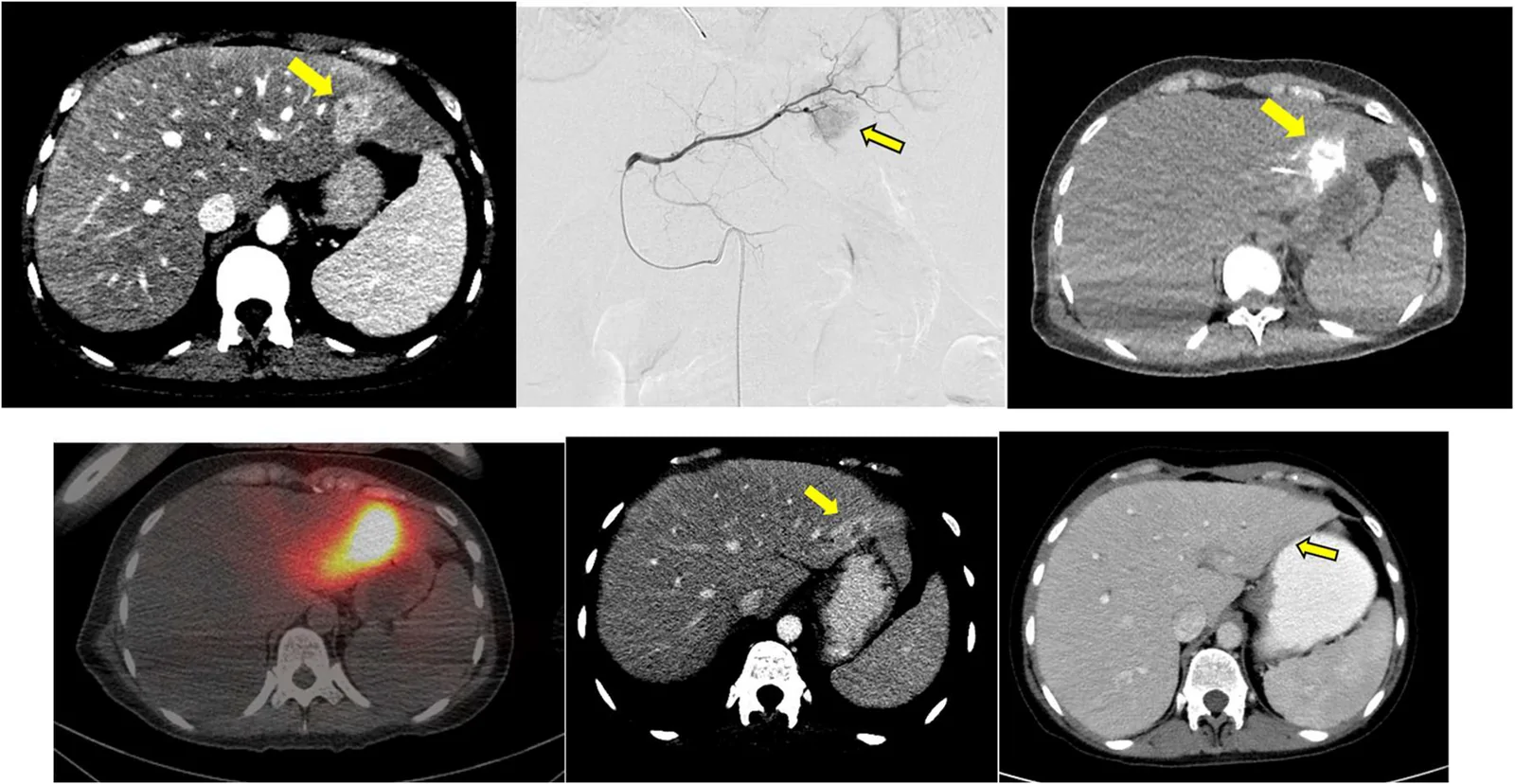
(**a**) Contrast-enhanced CT shows a segment 2/3 colorectal cancer liver metastasis measuring 3.5 cm. (**b**) Digital subtraction angiography of the left hepatic artery demonstrated tumor blush. (**c**) Intraoperative angio-CT demonstrated tumor coverage with selective catheterization. (**d**) Post-Y90 SPECT/CT demonstrated adequate uptake. (**e**) Follow-up CT at 4 months showed tumor shrinkage. (**f**) Follow-up CT at 6 months showed complete response

**Fig. 4:**
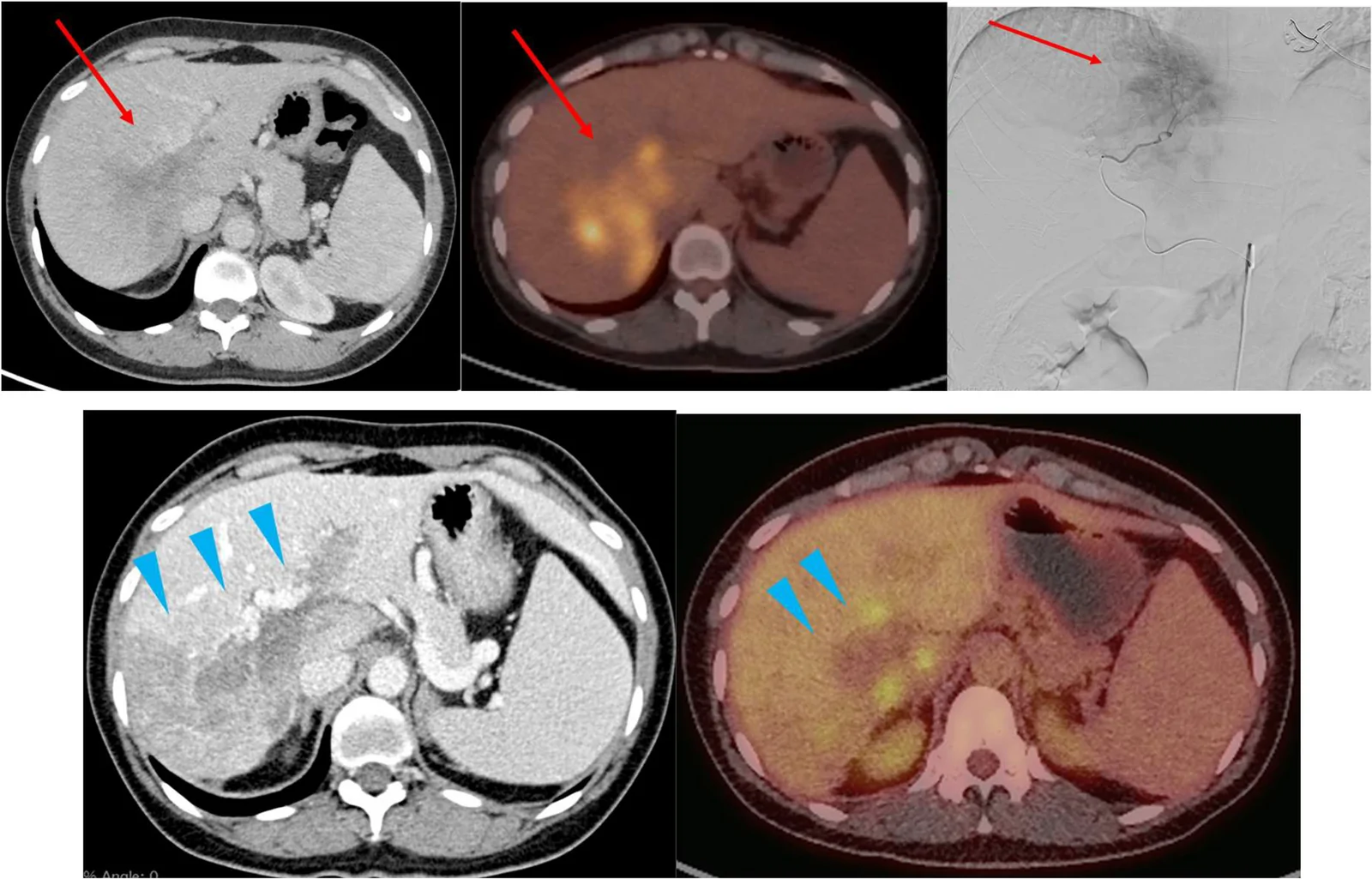
(**a**) and (**b**) Contrast enhanced CT and PET/CT demonstrated FDG-avid colorectal cancer liver metastasis. (**c**) Radiation lobectomy was performed via the right hepatic artery with boost dose to segment 8 and caudate branches. (**d**) Post embolization contrast enhanced CT showed treatment effect in the right hepatic lobe. (**e**) PET/CT showed reduced FDG avidity after radioembolization

**Fig. 5:**
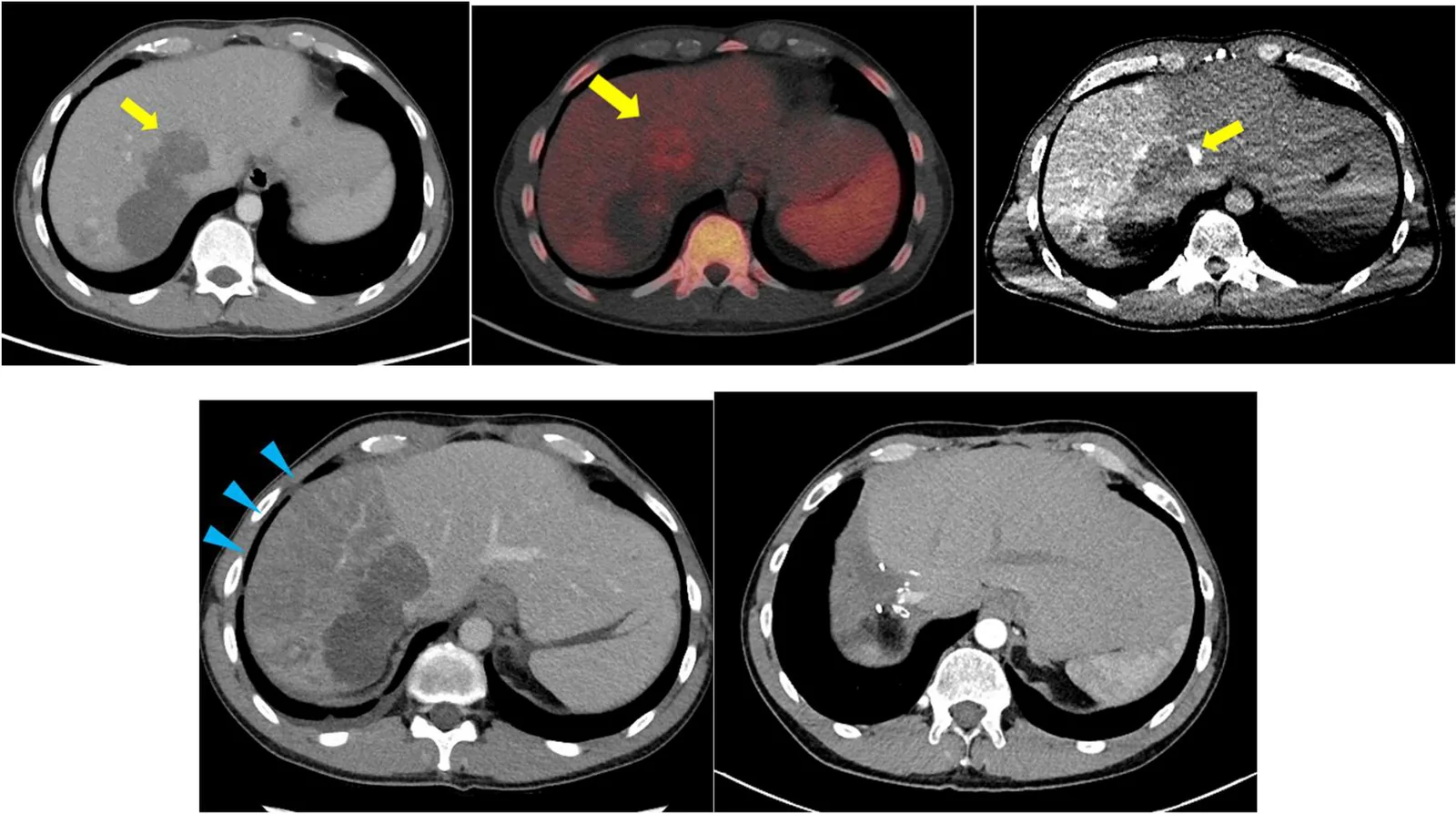
(**a**) and (**b**) Colorectal cancer with liver metastasis treated with systemic therapy presents with residual enhancing lesion in segment 4 and FDG avidity on PET/CT. (**c**) In order to achieve disease control and promote contralateral left lobe hypertrophy for consideration of future resection, radiation lobectomy was performed. Angio-CT showed coverage of the segment 4 tumor and the right lobe. (**d**) Post-treatment CT at 2 months showed treatment effects in the right hepatic lobe (blue arrows), as well as left hepatic lobe hypertrophy. (**e**) The patient underwent right hepatectomy

For patients with larger or multi-segment hepatic disease not amenable to segmentectomy alone, radiation lobectomy delivers Y-90 to an entire hepatic lobe to cover a larger, tumor-bearing volume to achieve disease control (Fig. 2). Additionally, this method also induces compensatory hypertrophy of the contralateral future liver remnant (FLR), functioning as a bridge to resection rather than a standalone ablative endpoint (Fig. 3) [44]. The technique has since evolved into modified radiation lobectomy (mRL), which combines an ablative dose to the tumor-bearing segments with a lower, and a lower lobectomy-level dose to the remaining non-tumor-bearing segments of the same lobe, achieving tumor control and FLR hypertrophy in a single treatment session, rather than sequential procedures [45]. This modified approach was recently characterized in detail by Yu et al. in a single institution series of 19 patients with right-sided unresectable primary hepatic tumors (13 HCC, 4 cholangiocarcinoma, 2 mixed HCC-cholangiocarcinoma) treated with either segmentectomy-plus-lobectomy or double-segmental approach [46]. Although this cohort was not CRLM-specific, the report provides one of the most granular technical descriptions of mRL dosing and FLR response kinetics that is directly applicable to liver-dominant CRLM (Figs. 4 and 5).

FLR hypertrophy achieved with radiation lobectomy in CRLM specifically has been directly compared to portal vein embolization (PVE), the conventional method of inducing FLR hypertrophy. In a retrospective single-center cohort of 20 CRLM patients (10 PVE, 10 radiation lobectomy) using functional assessment via 99mTc-mebrofenin hepatobiliary scintigraphy, radiation lobectomy took twice as long to achieve sufficient FLR function compared to PVE (median 75 vs. 31 days, *P* = 0.002), but produced a comparable functional increase (58.6% vs. 51.1%, *P* = 0.940) and a significantly greater hepatic volume increase (65.7% vs. 36.8%, *P* = 0.049), with no difference in post-hepatectomy liver failure or resection margins [47]. This finding is corroborated by a broader systematic review and meta-analysis of PVE versus Y-90 radioembolization for FLR hypertrophy. This study found hypertrophy was significantly greater with PVE than Y-90 (mean difference 23.75%, 95% CI 12.02–35.48, *P* < 0.0001), though a major limitation was that Y-90 was not administered at a dose specifically for hypertrophy induction [48]. Taken together, these data position radiation lobectomy not as a direct substitute for PVE but as a complementary strategy particularly suited to patients who require simultaneous tumor control and hypertrophy induction. This approach would be appropriate, for instance, for patients with large or centrally located tumors where PVE alone would leave active disease untreated during the hypertrophy interval. Prospective, CRLM-specific studies directly comparing mRL approaches to PVE, with standardized dosing protocols and resection outcomes as primary endpoints, remain an important area for future investigation.

## Future Directions

The clinical trajectory of radioembolization for CRLM is moving along several convergent fronts: toward personalized multi-compartment dosimetry, treatment earlier in the disease course, optimized patient selection, combinations with systemic therapy, and the potential for curative-intent ablative strategies in metastatic disease.

### Combination with Immunotherapy

The immunomodulatory impacts of radiation are well-established [49–51]. Radiated tumor cells can enhance tumor antigen presentation and can activate tumor-specific immune responses, and locoregional radiation has been hypothesized to potentiate the efficacy of immune checkpoint inhibitors through these mechanisms [50]. The unique properties of TARE, delivering a highly localized, high-dose radiation exposure without the cumulative marrow toxicity of external beam radiotherapy, make it an attractive partner for checkpoint blockade in a disease that has historically been resistant to immunotherapy. Several early-phase trials are now testing this combination prospectively, including the iRE-C trial of durvalumab combined with Y-90 radioembolization in CRLM (NCT04108481) and a separate METEORITE trial evaluating Y-90 TARE with capecitabine and atezolizumab in liver-dominant mCRC (NCT06555133). Results from these trials may help determine whether radiation-primed immune activation can extend the benefit of TARE beyond hepatic disease control.

### Biomarker-Driven Patient Selection

Beyond the anatomic and functional criteria used in current practice, prespecified subgroup analyses from EPOCH suggest that molecular and clinical features may further refine patient selection. The PFS benefit of TARE was most pronounced in patients with KRAS-mutated tumors (HR 0.57), left-sided primary tumors (HR 0.65), tumor burden of 10–25% (HR 0.43), and three or fewer hepatic lesions (HR 0.33), with no discernable PFS benefit among patients with right-sided primary tumors [21]. This finding contrasts with first-line FOXFIRE, SIRFLOX, and FOXFIRE-Global studies, in which the addition of TARE was associated with an apparent OS benefit among patients with right-sided primary tumors despite no significant PFS difference [32]. These discordant findings suggest that the relationship between primary tumor sidedness and TARE benefit may depend on treatment setting and disease biology, and warrant further investigation before sidedness is incorporated into treatment selection. The prognostic importance of primary tumor sidedness is well established in mCRC [52], although its predictive value for TARE remains uncertain. Prospective validation of these signals, ideally alongside biomarkers such as lymphovascular invasion status identified in salvage-setting cohorts, will be necessary before biomarker-driven selection can be formally incorporated into treatment algorithms.

### Neoadjuvant and Downstaging Applications

Although SIRFLOX and the pooled FOXFIRE analysis did not demonstrate increased conversion-to-resection rates despite improved hepatic response with first-line TARE [31], interest has shifted toward evaluating radioembolization specifically as a neoadjuvant strategy in potentially resectable disease, rather than as an unselected adjunct to first-line chemotherapy. The recently initiated YRENE trial (NCT07406126) is evaluating neoadjuvant Y-90 radioembolization with immune marker profiling and personalized dosimetry in patients with potentially resectable CRLM, aiming to determine whether pre-operative hepatic disease control with systemic therapy and immune activation can improve downstaging and surgical outcomes. Defining the appropriate dose, timing, and target population for neoadjuvant use remains an open question distinct from the salvage- and second-line indications that are currently supported by the available evidence.

## Conclusion

Radioembolization has evolved from a salvage-only modality to an evidence-supported component of the multidisciplinary management of colorectal liver metastases. Rigorous patient selection, prioritizing those with preserved performance status metrics, limited hepatic burden, and adequate hepatic reserve, remain the most consequential determinants of response. Current evidence supports the second-line and later use in combination with radiosensitizing chemotherapy as the best-defined indication, while unselected first-line use cannot be justified on the basis of lack of survival benefit alone. The field is increasingly moving toward ablative, dosimetry-driven segmental approaches with curative intent in oligometastatic disease. Additionally, combined immunomodulatory therapy and biomarker-driven selection trials will be essential for defining the next generation of evidence. Until then, radioembolization is best delivered at experienced multidisciplinary centers where patient selection, procedural expertise, and dosimetric optimization are systematically applied.

## Data Availability

No datasets were generated or analysed during the current study.
